# Supplementary material for: Fine‐tuning quantitative agronomic traits by manipulating gene copy number in rice
Source: New Phytol. 2026 May 13;251(4):1609–16. doi: 10.1111/nph.71258 (PMC13373809; doi:10.1111/nph.71258)
Supplement: Supplementary file 2 — Fig. S1 Diagram of the method used to determine OsMADS18 copy number by quantitative PCR in rice (Oryza sativa). Fig. S2 Tandem duplication of the OsMADS18 genomic region on chromosome 7 in rice (Oryza sativa) cv Hitomebore. Fig. S3 Genome browser views of the OsMADS18 genomic region on chromosome 7 in rice (Oryza sativa). Fig. S4 OsMADS18 copy number variation in T0 plants and progeny as determined by quantitative PCR in rice (Oryza sativa). Fig. S5 Genomic structure of the OsMADS18 locus in IRGSP‐1.0 and copy number variation (CNV)‐edited lines in rice (Oryza sativa). Fig. S6 Effects of OsMADS18 copy number variation (CNV) on other agronomic traits in rice (Oryza sativa). Fig. S7 Effects of OsMADS18 copy number variation (CNV) on agronomic traits in an independent experiment in rice (Oryza sativa). Fig. S8 Effects of OsMADS18 copy number variation (CNV) on tiller number in rice (Oryza sativa). Table S1 List of primers used for rice (Oryza sativa) in this study. Table S2 List of sequencing data and corresponding Sequence Read Archive (SRA) accession numbers for rice (Oryza sativa). Please note: Wiley is not responsible for the content or functionality of any Supporting Information supplied by the authors. Any queries (other than missing material) should be directed to the New Phytologist Central Office. [file NPH-251-1609-s001.docx]

## *New Phytologist* Supporting Information

**Article title:** Fine-tuning quantitative agronomic traits by manipulating gene copy number in rice

**Authors:** Chihiro Nomura, Hiroyuki Kanzaki, Eiko Kanzaki, Motoki Shimizu, Kaori Oikawa, Hiroe Utsushi, Kazue Ito, Yusaku Sugimura, Ryohei Terauchi, Akira Abe

**Article acceptance date:** 21 April 2026

The following Supporting Information is available for this article:

**Fig. S1** Diagram of the method used to determine *OsMADS18* copy number by quantitative PCR in rice (*Oryza sativa*).

**Fig. S2** Tandem duplication of the *OsMADS18* genomic region on chromosome 7 in rice (*Oryza sativa*) cv. Hitomebore.

**Fig. S3** Genome browser views of the *OsMADS18* genomic region on chromosome 7 in rice (*Oryza sativa*).

**Fig. S4** *OsMADS18* copy number variation in T_0_ plants and progeny as determined by quantitative PCR in rice (*Oryza sativa*).

**Fig. S5** Genomic structure of the *OsMADS18* locus in IRGSP-1.0 and copy number variation (CNV)-edited lines in rice (*Oryza sativa*).

**Fig. S6** Effects of *OsMADS18* copy number variation (CNV) on other agronomic traits in rice (*Oryza sativa*).

**Fig. S7** Effects of *OsMADS18* copy number variation (CNV) on agronomic traits in an independent experiment in rice (*Oryza sativa*).

**Fig. S8** Effects of *OsMADS18* copy number variation (CNV) on tiller number in rice (*Oryza sativa*).

**Table S1** List of primers used for rice (*Oryza sativa*) in this study.

**Table S2** List of sequencing data and corresponding Sequence Read Archive (SRA) accession numbers for rice (*Oryza sativa*).

**
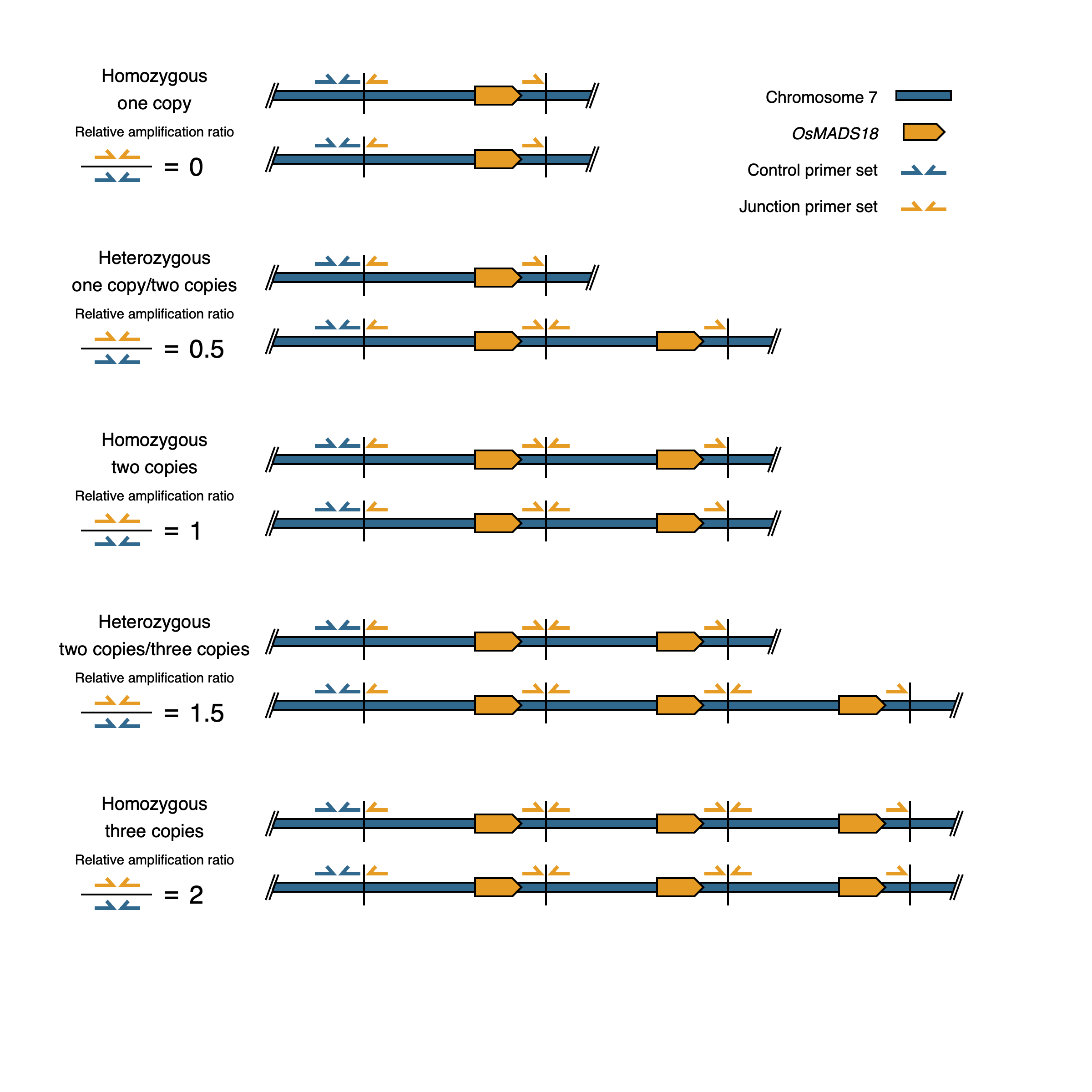
Fig. S1** Diagram of the method used to determine *OsMADS18* copy number by quantitative PCR in rice (*Oryza sativa*). When the relative amplification ratio of Hitomebore (homozygous for two copies of *OsMADS18*) is set to 1, the values for different genotypes are as follows: homozygous for one copy, 0; heterozygous for one and two copies, 0.5; heterozygous for two and three copies, 1.5; homozygous for three copies, 2.

**Fig. S2** Tandem duplication of the *OsMADS18* genomic region on chromosome 7 in rice (*Oryza sativa*) cv. Hitomebore. (a, b) Dot matrix plots of the *OsMADS18* genomic region between Hitomebore and IRGSP-1.0 (a) and between Sasanishiki and IRGSP-1.0 (b). Grid lines are spaced at 0.03-Mb intervals. (c) Relative sequencing depth of Hitomebore (the target) to Sasanishiki (the control) using Illumina short reads around *OsMADS18*. A sliding window with a window size of 5,000 bp and a step size of 500 bp was used. The orange shaded area marks the position of *OsMADS18* for (a–c). (d) One single-nucleotide polymorphism and three insertion/deletion polymorphisms were observed in duplicated region 2 relative to duplicated region 1.

**(a)**

**(b)**

**(c)**

**
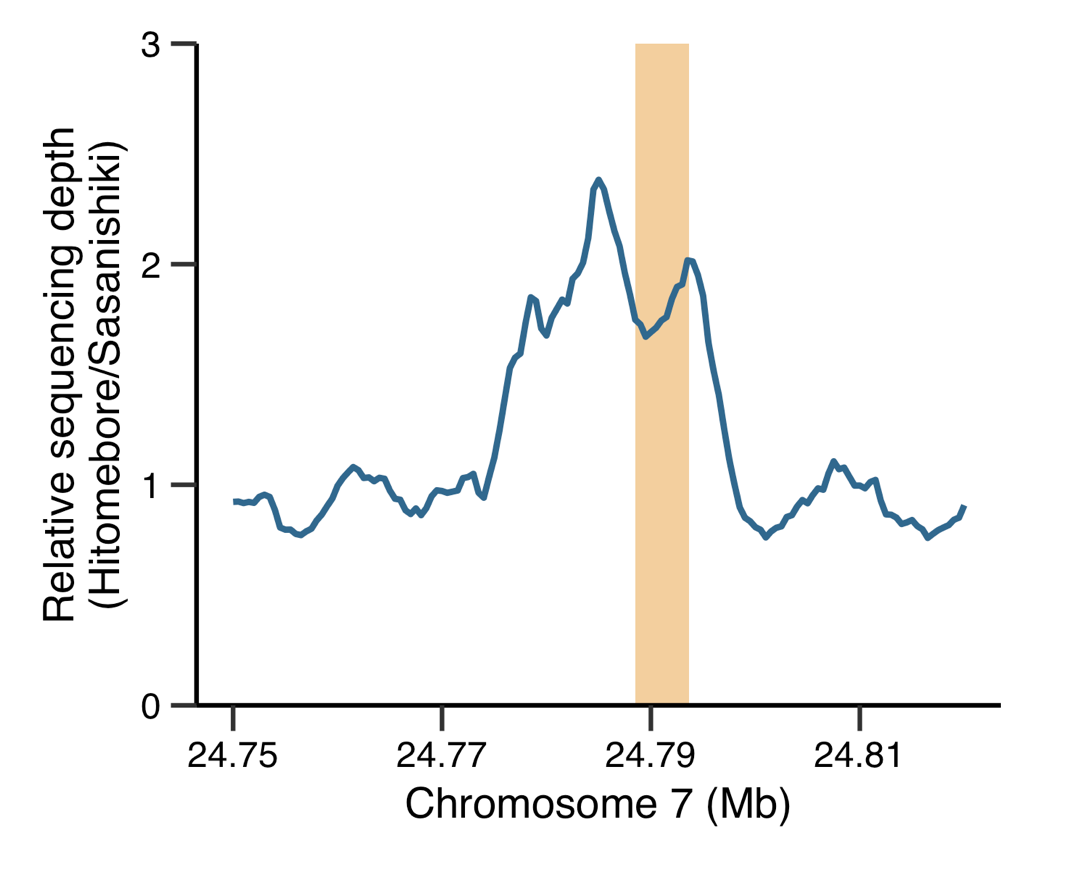
**

**
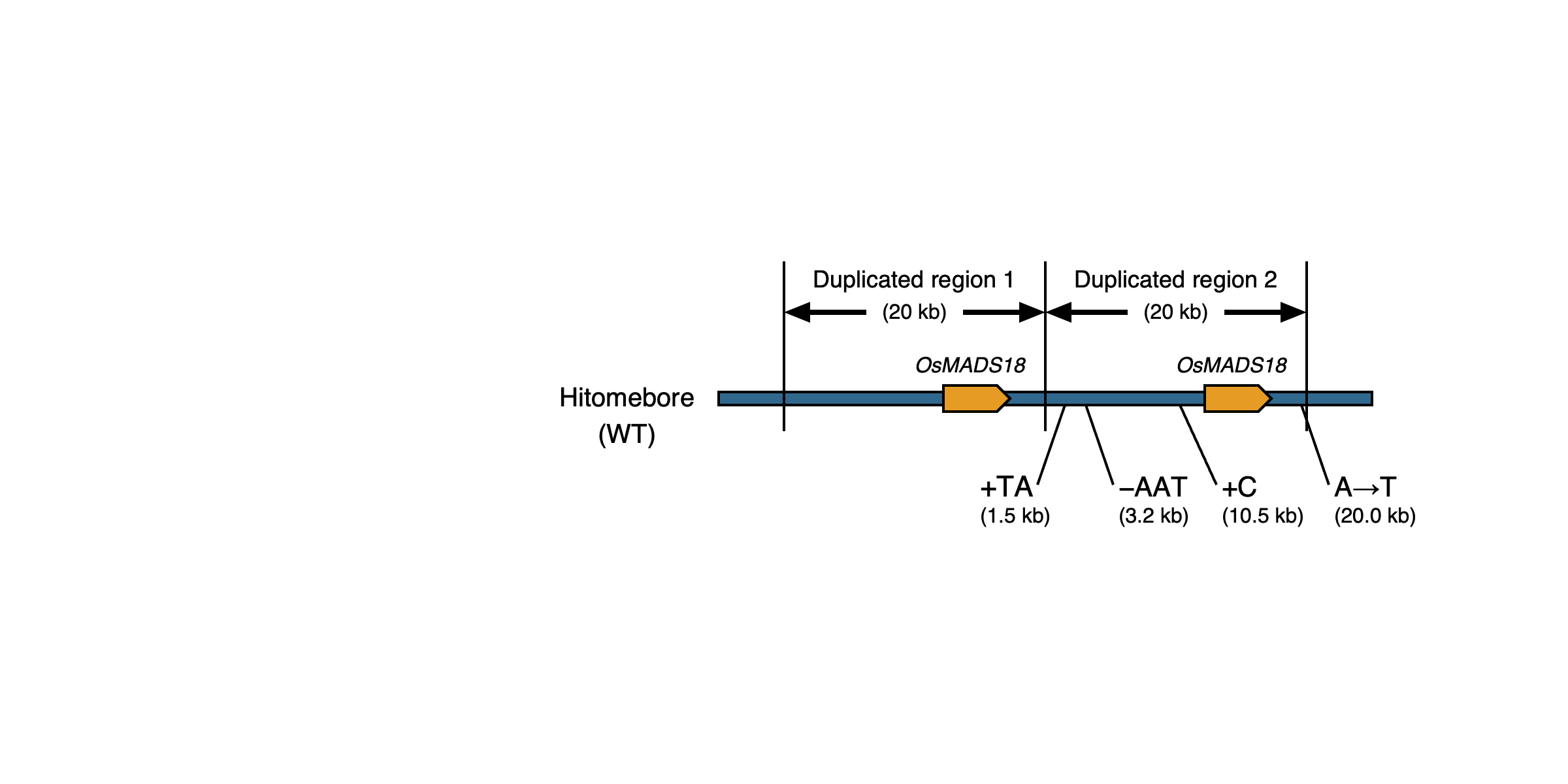
**

**(d)**

**Fig. S3** Genome browser views of the *OsMADS18* genomic region on chromosome 7 in rice (*Oryza sativa*). (a) Integrative Genomics Viewer (IGV) image of a 30-kb window in Hitomebore. The red horizontal bar indicates the duplicated segment. (b) IGV image of a 30-kb window in Sasanishiki. (c) JBrowse image of the duplicated *OsMADS18* region in the Rice Annotation Project Database (RAP-DB).

**(a)**


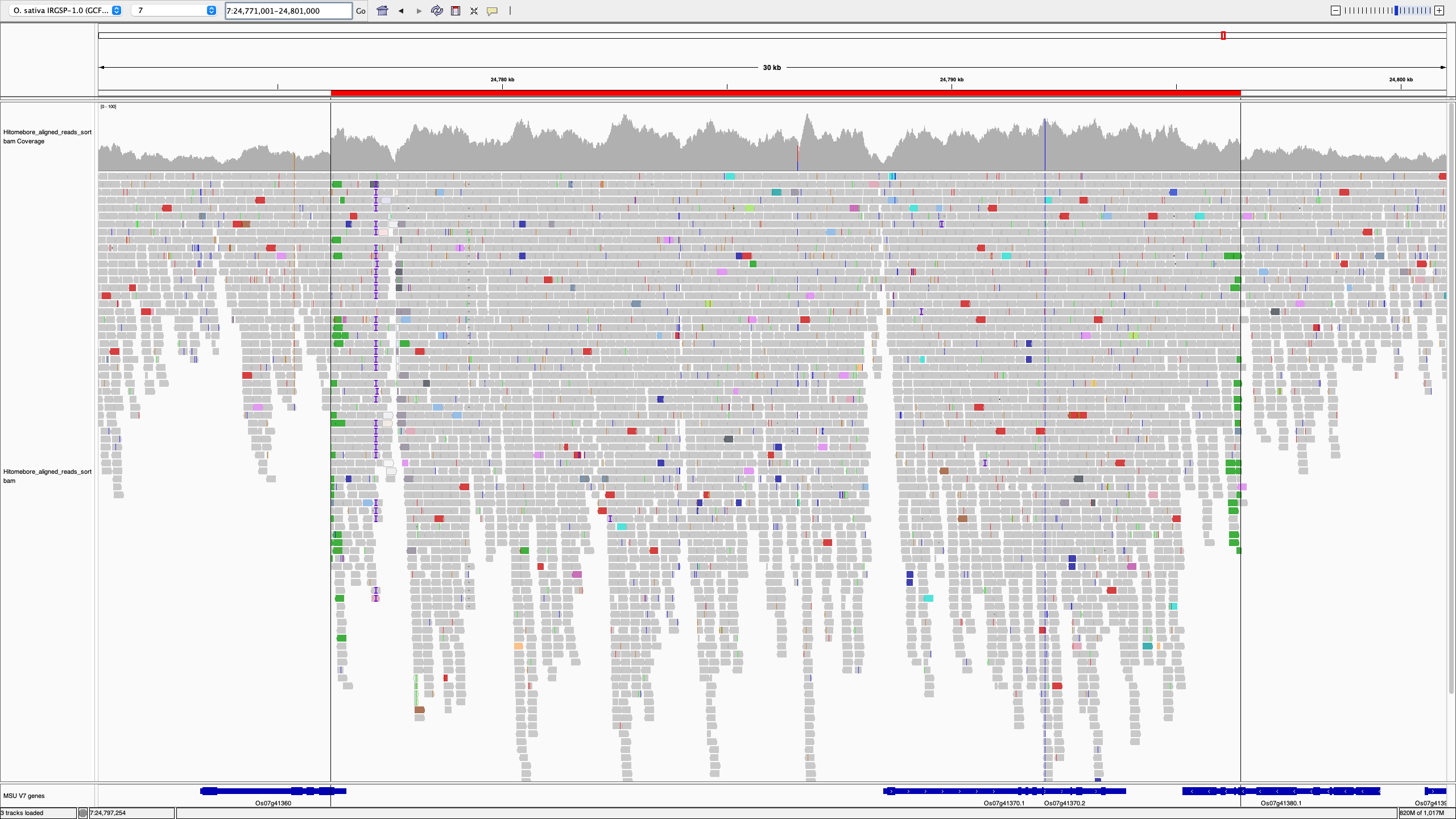


***OsMADS18***

**(b)**

**
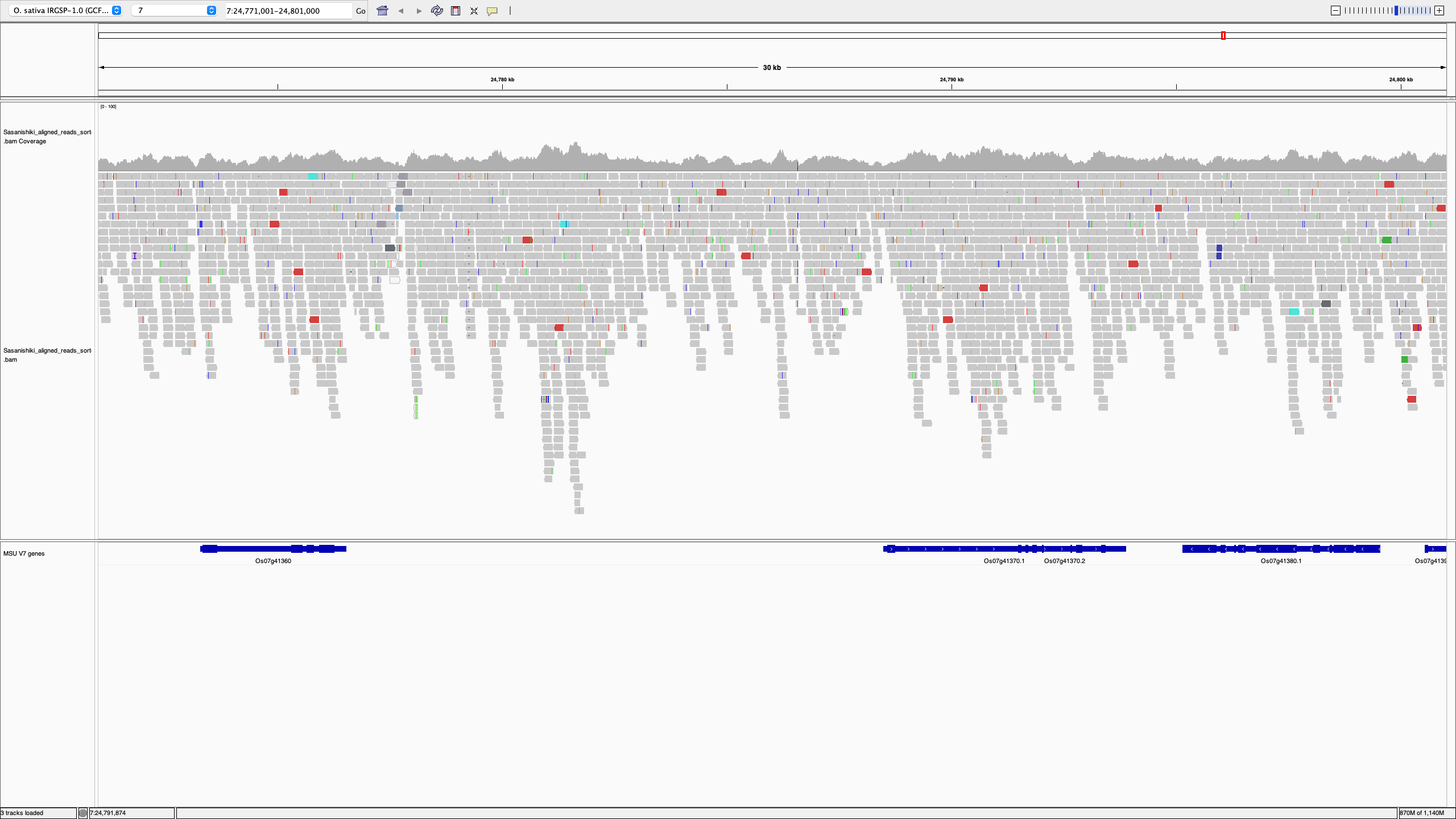
**

***OsMADS18***

**(c)**

**
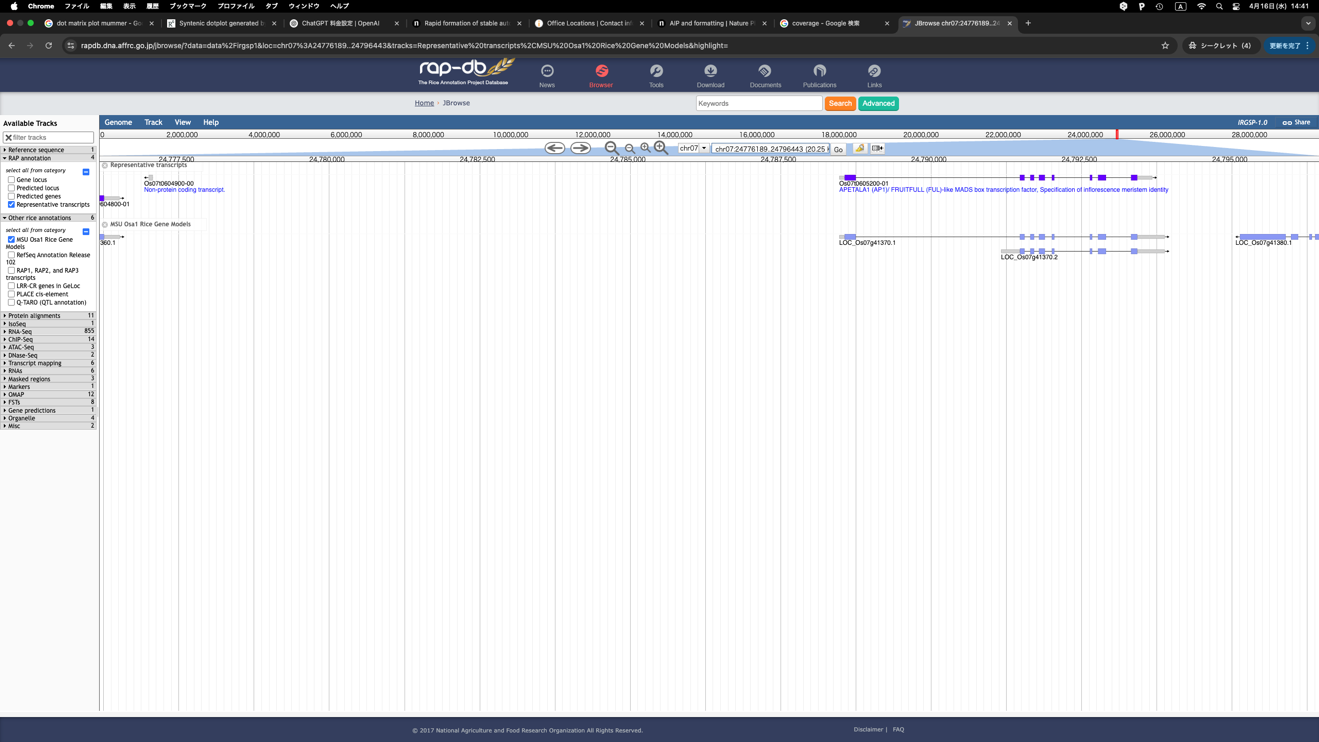
**

***OsMADS18***

**Fig. S4** *OsMADS18* copy number variation in T_0_ plants and progeny as determined by quantitative PCR in rice (*Oryza sativa*). (a) Frequency distribution of the relative amplification ratio for copy number determination in transgenic plants (T_0_). (b) Summary table showing the numbers and percentages of T_0_ plants with estimated copy number alterations. T_0_ plants derived from different calli or from the same callus with different culture periods were counted as independent lines. The relative amplification ratio was used to classify heterozygous plants as follows: heterozygous for one copy/two copies, 0.3 < *x* < 0.7; heterozygous for two copies/three copies, 1.3 < *x* < 1.7; *x* represents the relative amplification ratio. (c) Frequency distribution of progeny (*n* = 400) derived from plants estimated to be heterozygous for one copy/two copies (relative amplification ratio is ~0.5). (d) Frequency distribution of progeny (*n* = 12) derived from plants estimated to be heterozygous for two copies/three copies (relative amplification ratio is ~1.5).

**(b)**

**(c)**

**(a)**

**(d)**

**Fig. S5** Genomic structure of the *OsMADS18* locus in IRGSP-1.0 and copy number variation (CNV)-edited lines in rice (*Oryza sativa*). (a, b) Dot matrix plots comparing the *OsMADS18* genomic region in the Nipponbare reference genome (IRGSP-1.0) to that in plants with one homozygous copy (1 CNV) (a) or three homozygous copies (3 CNV) (b) of *OsMADS18*. Grid lines are spaced at 0.03-Mb intervals. The orange shaded area marks the position of *OsMADS18*.

**(a)**

**(b)**

**Fig. S6** Effects of *OsMADS18* copy number variation (CNV) on other agronomic traits in rice (*Oryza sativa*). (a) Representative photograph of overall plant architecture. (b) Leaf blade length of the leaf just below the flag leaf, compared among individuals with the same total number of leaves (12 leaves) on the main stem (*n* = 29, 35, and 29 for 1, 2, and 3 CNV, respectively). (c) Days to heading (*n* = 30, 35, and 36 for 1, 2, and 3 CNV, respectively). (d) Leaf number (*n* = 30, 35, and 36 for 1, 2, and 3 CNV, respectively). “1 CNV,” “2 CNV,” and “3 CNV” refer to plants with one, two, and three homozygous copies of *OsMADS18*, respectively. Different lowercase letters indicate significant differences (*P* < 0.05) based on the Tukey–Kramer test in panels (b) and (c). No significant differences (adjusted *P* > 0.05) were detected in any pairwise comparison among the three genotypes based on Fisher’s exact test with Holm–Bonferroni correction in panel (d). Violin plots were generated using kernel density estimation with a smoothing adjustment factor of 1.5. Boxplots represent the first and third quartiles (the lower and upper hinges, respectively), the median (the middle horizontal line), and the range from the hinges to the smallest and largest observations within 1.5 times the interquartile range (the bottom and top whiskers, respectively). All data points, including outliers in the boxplot, are displayed as dots in the dot plot.

a

a

a

a

b

c

**(b)**

**(a)**

**(c)**

1 CNV

2 CNV

3 CNV

**(d)**

**Fig. S7** Effects of *OsMADS18* copy number variation (CNV) on agronomic traits in an independent experiment in rice (*Oryza sativa*). (a, b) Flag leaf blade length, compared among individuals with the same total number of leaves on the main stem: 12 leaves in panel (a) (*n* = 42, 31, and 5 for 1, 2, and 3 CNV, respectively) and 11 leaves in panel (b) (*n* = 6, 17, and 42 for 1, 2, and 3 CNV, respectively). (c) Culm length. (d) Days to heading. (e) Leaf number. (f) Number of primary branches per panicle. (g) Number of secondary branches per panicle. (h) Number of spikelets on primary branches per panicle. (i) Number of spikelets on secondary branches per panicle. (j) Number of spikelets per panicle. (k) Mean single-grain weight. “1 CNV,” “2 CNV,” and “3 CNV” refer to plants with one, two, and three homozygous copies of *OsMADS18*, respectively. For all panels except (a) and (b), *n* = 48, 48, and 47 for 1, 2, and 3 CNV, respectively. Different lowercase letters indicate significant differences (*P* < 0.05) based on the Tukey–Kramer test in panels (a–d) and (f–k). Significant differences (adjusted *P* < 0.05) were detected in all pairwise comparisons based on Fisher’s exact test with Holm–Bonferroni correction in panel (e). Violin plots were generated using kernel density estimation with a smoothing adjustment factor of 1.5. Boxplots represent the first and third quartiles (the lower and upper hinges, respectively), the median (the middle horizontal line), and the range from the hinges to the smallest and largest observations within 1.5 times the interquartile range (the bottom and top whiskers, respectively). All data points, including outliers in the boxplot, are displayed as dots in the dot plot.

**(d)**

**(c)**

**(b)**

**(a)**

a

b

a

c

b

a

a

a

b

a

b

b

**(e)**

**(f)**

**(i)**

**(h)**

**(g)**

a

b

a

b

b

a

b

b

a

b

b

b

c

b

a

b

a

b

**(k)**

**(j)**

**Fig. S8** Effects of *OsMADS18* copy number variation (CNV) on tiller number in rice (*Oryza sativa*). Tiller number per plant was counted at 56 days after sowing (*n* = 37, 37, and 35 for 1, 2, and 3 CNV, respectively). “1 CNV,” “2 CNV,” and “3 CNV” refer to plants with one, two, and three homozygous copies of *OsMADS18*, respectively. Different lowercase letters indicate significant differences (*P* < 0.05) based on the Tukey–Kramer test. Boxplots represent the first and third quartiles (the lower and upper hinges, respectively), the median (the middle horizontal line), and the range from the hinges to the smallest and largest observations within 1.5 times the interquartile range (the bottom and top whiskers, respectively). All data points, including outliers in the boxplot, are displayed as dots in the dot plot.

a

a

a

**Table S1** List of primers used for rice (*Oryza sativa*) in this study.

**Table S2** List of sequencing data and corresponding Sequence Read Archive (SRA) accession numbers for rice (*Oryza sativa*).
